# Acute Effects of Particulate Air Pollution on Ischemic Heart Disease Hospitalizations in Shanghai, China

**DOI:** 10.3390/ijerph14020168

**Published:** 2017-02-09

**Authors:** Anyang Xu, Zhe Mu, Bo Jiang, Wei Wang, Han Yu, Lijuan Zhang, Jue Li

**Affiliations:** 1 Department of Prevention, School of Medicine, Tongji University, Shanghai 200092, China; Anyang_7140@163.com (A.X.); 13918850310@163.com (B.J.); wangwei91@tongji.edu.cn (W.W.); 0705jessica@tongji.edu.cn (H.Y.); 2 Heart, Lung and Blood Vessel Center, Tongji University School of Medicine, Shanghai 200092, China; 3 Shanghai Key Laboratory of Meteorology and Health, Shanghai Meteorological Service, Shanghai 200000, China; muzhe123456@126.com

**Keywords:** particulate matter, ischemic heart disease, hospitalizations

## Abstract

*Background:* Air pollution has been demonstrated to be a major risk factor for the development of cardiovascular and respiratory diseases worldwide. This study examines the relationship between the exposure to fine particulate matter (PM) and patient hospitalizations as a result of ischemic heart disease (IHD) during 2013–2014 in Shanghai, China. *Methods:* Daily IHD hospitalization data were acquired from the Shanghai Health Insurance Bureau (SHIB) from 1 January 2013 to 21 December 2014. Daily average concentrations of air pollution as well as meteorological data were obtained from the database of Shanghai Environmental Monitoring Center (SEMC) during the same time period, and all data were analyzed using standard epidemiological methodology. Generalized linear model (GLM) adjusted for time trends, weather conditions, and medical insurance policy was used to estimate the immediate and delayed effects of PMs on IHD hospitalizations, and the effects of PMs were also examined based on gender, age group and seasonal variation. *Results:* A total of 188,198 IHD hospitalizations were recorded during 2013–2014 in Shanghai, China. During this period, the average concentrations of the fine particulate matter with aerodynamic diameter of <10 μm (PM_10_) and ≤2.5 (PM_2.5_) were 76 µg/m^3^ and 56.3 µg/m^3^, respectively. The effect of PMs was strongest on days when a 10 μg/m^3^ increment increase of PM_2.5_ and PM_10_, which coincided with an increase in IHD hospitalizations by 0.25% (95% CI: 0.10%, 0.39%) and 0.57% (95% CI: 0.46%, 0.68%), respectively. Furthermore, the effect of PMs was significantly greater in males and people between 41 and 65 years old. *Conclusions*: Hospitalizations of IHD was strongly associated with short-term exposure to high levels of PM_10_ and PM_2.5_ during 2013–2014 in Shanghai, China.

## 1. Introduction

Ischemic heart disease (IHD) is characterized by myocardial ischemia due to the narrowing of the coronary vessels which supply blood to the heart [1]. IHD is one of the most common causes of deaths worldwide, representing 12.7% of the total global mortality in 2008, as well as being the second leading cause of premature mortality in 2010, with a disability-adjusted life years (DALYs) lost of 17.9 million person-years, and a potential years of life lost (PYLL) of 16.1 million person-years [2].

A number of risk factors for IHD have been identified over the past few years, including age, gender, hypertension, obesity and smoking [3]. Particulate air pollution has also been associated with the increased occurrence of IHD hospital admissions, including both types of fine particulate matters with aerodynamic diameters of <10 μm (PM_10_) and ≤2.5 (PM_2.5_) [4,5,6]. Several epidemiological studies have indicated that short-term increases in ambient air pollutants may increase the mortality, hospital admissions, and emergency visits for patients with cardiovascular diseases [7,8,9,10]. A report by the World Health Organization (WHO) indicated that particulate matters (PMs) accounted for as much as 9.4% of all kinds of IHD [11], which suggested that particulate air pollution was one of the world’s leading environmental health risks.

There have been numerous studies in China which focused on the relationship between PMs and heart disease in heavy industrial cities with high levels of air pollution, such as Beijing, Shenyang and Tianjin [12,13,14], however few reports have examined the relationship between the effect of PMs and public health in areas with relatively lower concentrations of PM. Shanghai is one of the highest populated metropolises in China, and is affected by slight to moderate levels of air pollution [15], and thus examining the effects of PMs on the health of local inhabitants is crucial. In this study, we performed a time-series study to investigate the acute effects of ambient particulate matter on IHD hospitalizations among people aged over 40 in Shanghai, China.

## 2. Materials and Methods

### 2.1. Health Data

Health data were obtained from Shanghai Health Insurance Bureau (SHIB) from 1 January 2013 to 31 December 2014, which provided compulsory universal health insurance to 89.1% of the permanent residents in Shanghai [16]. The remaining residents were either covered by private health insurance or had no health insurance registered by the SHIB. Computerized records of hospitalizations were maintained at each contracted hospital and sent to the SHIB through an internal computer network [17]. The primary classification of daily IHDs were based on the International Classification of Disease, 10th revision for Ischemic Heart Disease (ICD10: I20–I25). Daily IHD counts were stratified by gender and age groups (41–65, 66–85, and >85 year-old). The study protocol was approved by the Institutional Review Board at Tongji University (2013YXY14). Health information was provided to us without identification of individual patients, and no contact was made with any patients in this study.

### 2.2. Air Pollution and Meteorological Data

**Figure 1:**
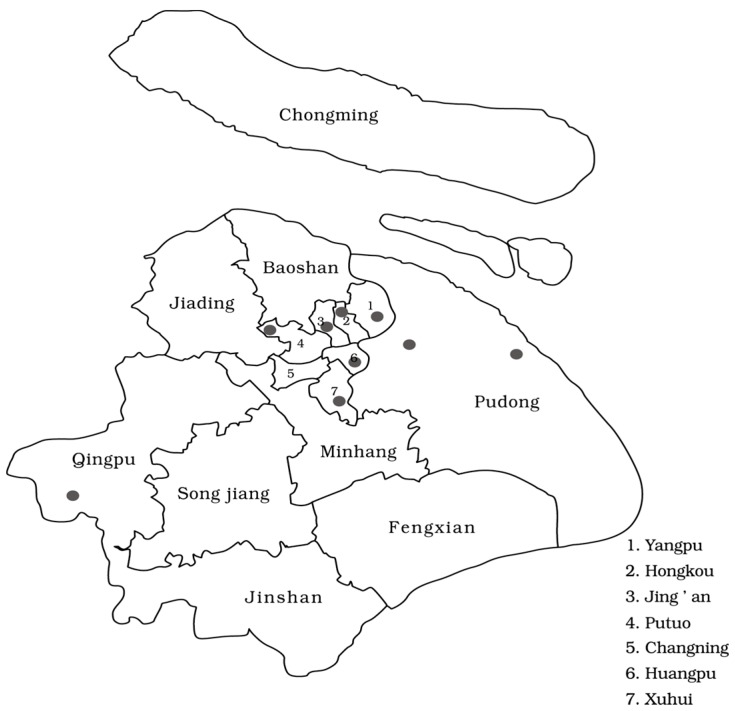
Location of the nine air quality monitoring stations in Shanghai.

Air pollution and meteorological data were obtained from the database of Shanghai Environmental Monitoring Center (SEMC) Shanghai, China from 1 January 2013 to 31 December 2014. The SEMC database included 24-h average measurements of PM_10_ and PM_2.5_ concentration, as well as the daily maximum OZONE figure based on an 8-h running mean. All data used in this study were based on the average assessment of nine air quality monitoring stations in Shanghai (Figure 1), located in Putuo, Yangpu, Huangpu ,Qingpu, Hongkou, Xuhui, Jing’an, and Pudong New Area. These stations are mandated to be located away from major roads, industrial sources, buildings, or residential sources of emissions including coal, waste, or oil. Thus, our results reflect the background urban air pollution level in Shanghai rather than local sources of pollution such as traffic or industrial combustion [18]. Additional parameters including daily mean temperature and relative humidity (RH) were also obtained from the Shanghai Meteorological Bureau.

### 2.3. Statistical Analysis

SPSS 19.0 software (2010 SPSS Inc., Chicago, IL, USA) was used for data input, and statistical software R (version 3.2.3, R Foundation for Statistical Computing, Vienna, Austria) was used for data analysis. The baseline data were presented as mean ± standard deviation (SD) for continuous variables, *p* < 0.05 was considered as statistically significant.

A generalized linear model (GLM) was used to analyze the association between air pollutants and daily hospitalizations as a result of IHD [19]. Daily hospitalizations typically followed an over-dispersed Poisson distribution, and therefore we used quasi-Poisson regression in this model. Multivariable regression model was used to adjust for covariates, including: (1) A natural cubic smooth function of calendar time with seven degrees of freedom (df) per year [20], to exclude unmeasured long-term and seasonal trends; (2) The average temperature of the five previous days (lag 05) to cover for the effects during the entire time period, and the current day-to-day relative humidity (df = 3) to control for the potential nonlinear confounding effects of weather conditions; (3) Three indicator variables were used for the day of the week, public holiday and medical insurance policy, respectively.

For sensitivity analysis, we examined the impacts of lag 0–lag 7 and lag 01–lag 03 on PM concentration. A lag of 0 day (lag 0) corresponded to the current-day pollutant concentration, and a lag of 1 day (lag 1) referred to the previous-day concentration; Lag 01 corresponded to the 2-day moving average of pollutant concentration from the current and previous day. Preliminary results showed that the effect of lag 0 was the strongest, hence this study adopted lag 0 for the majority of the analyses.

To test the stability effects of PM on IHD hospitalizations, we also included OZONE assessment with the current-day concentration (lag 0). In addition, data were stratified by age, gender and cold season (from November to April).

## 3. Results

### 3.1. Descriptive Analysis

A total of 188,198 patient IHD hospitalizations were recorded, of which 51.8% were female and 51.1% were aged between 65 and 85. Across all seasons, there was an average of 258 hospitalizations per day, compared to an average of 268 hospitalizations per day in the cold season (Table 1). The distribution of daily air pollution concentrations and weather variables in Shanghai from 1 January 2013 to 31 December 2014 were summarized in (Table 2). Across all seasons, the daily mean concentrations of air pollutants were 76 µg/m^3^ for PM_10_, 56.3 µg/m^3^ for PM_2.5_ and 101.5 µg/m^3^ for OZONE. The daily average temperature and humidity were 14.1 °C and 70.2%, respectively, which reflected the subtropical climate in Shanghai. In the cold season, the daily mean concentrations for PM_10_ and PM_2.5_ were 70 µg/m^3^ and 91.9 µg/m^3^, respectively, while the daily average temperature was 6.3 °C.

### 3.2. Estimated Effects of Air Pollutants

**Figure 2:**
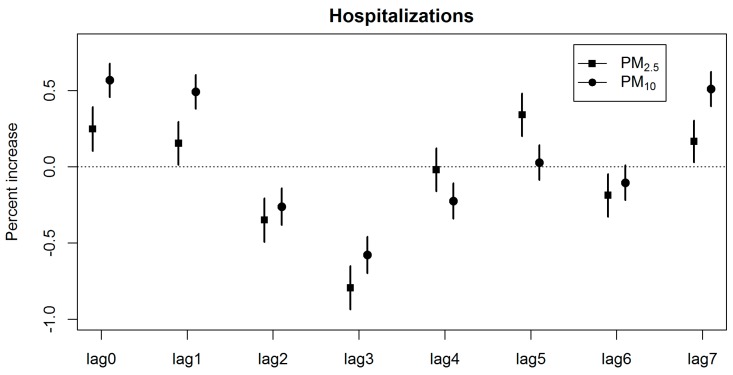
Percentage increase in IHD hospitalizations (95% CI) associated with a 10 μg/m^3^ increase in PM_2.5_ and PM_10_ concentrations from lag 0 to lag 7 across all seasons.

The linear effect of each pollutant at different time lags was evaluated using the single pollutant model after controlling for various factors including temperature, atmospheric pressure and relative humidity. IHD hospitalizations were associated with a 10 μg/m^3^ increase in PM_2.5_ and PM_10_ concentrations from lag 0 to lag 7 (Figure 2).

This corresponded to an increase in IHD hospitalizations by 0.25% (95% CI: 0.10%, 0.39%) and 0.57% (95% CI: 0.46%, 0.68%), respectively for PM_2.5_ and PM_10_. The single lag effect of PM_2.5_ and PM_10_ on IHD hospitalizations was most notable at lag 0. In addition, IHD hospitalizations were also associated with an increase in PM_2.5_ at lag 1, lag 5 and lag 7, and with PM_10_ at lag 1 and lag 7.

Table 3 showed the percentage change in IHD hospitalizations associated with a 10 µg/m^3^ increase in PM concentrations in both the cold season and across all seasons. A positive effect of PM was observed in each group except the >85 year-old group. Furthermore, a greater effect was observed among males and people aged between 40 and 65, indicating that gender and age may have an effect on the association between PM and IHD admission rate.

**Figure 3:**
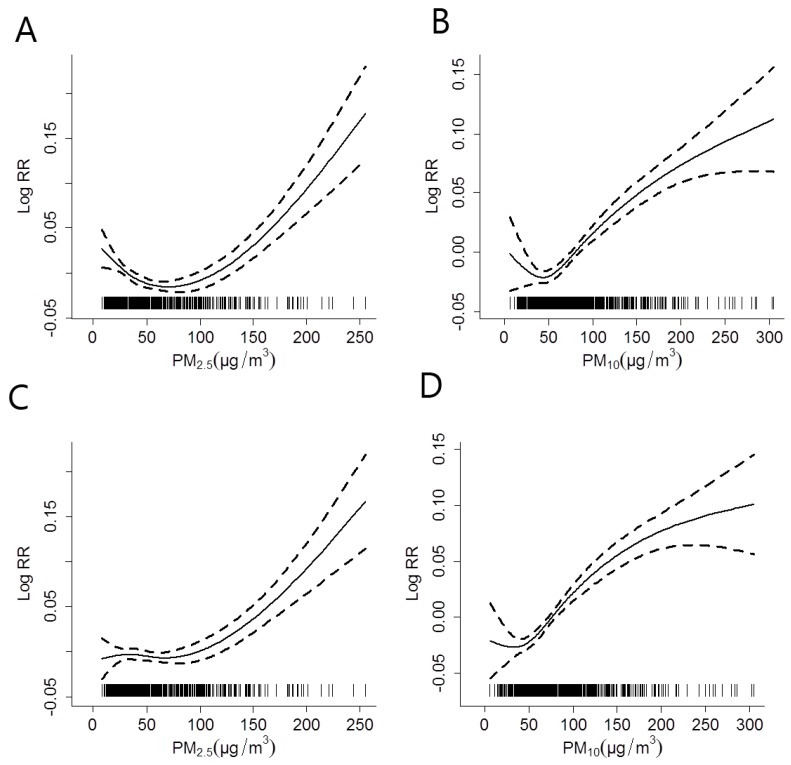
The concentration-response relationships for PM_2.5_ and PM_10_ with IHD hospitalizations at lag 0 across all seasons, after adjusting for the effect of OZONE (**C**,**D**) or without adjustment (**A**,**B**). The X-axis represents the PM concentrations (µg/m^3^) on the concurrent day. The Y-axis represents the log-relative risk on the IHD hospitalizations. The solid line indicates the estimated mean change in the log-relative risk, while the dotted lines represent the 95% CI of the estimates.

**Figure 4:**
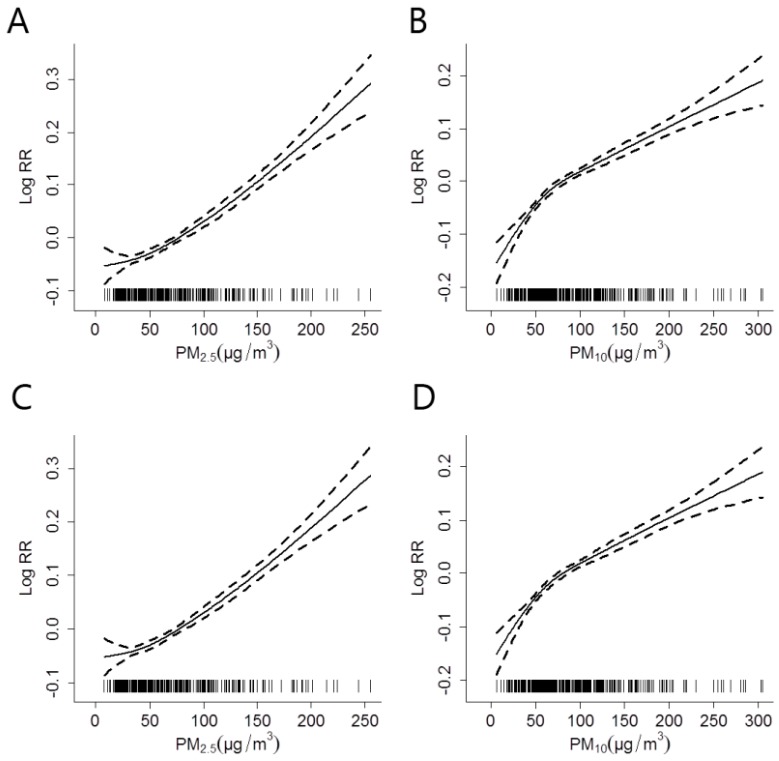
The concentration-response relationships for PM_2.5_ and PM_10_ with IHD hospitalizations at lag 0 in the cold season, after adjusting for the effect of OZONE (**C**,**D**) or without adjustment (**A**,**B**). The X-axis represents the PM concentrations (µg/m^3^) on the concurrent day. The Y-axis represents the log-relative risk on the IHD hospitalizations. The solid line indicates the estimated mean change in the log-relative risk, while the dotted lines represent the 95% CI of the estimates.

The concentration-response relationships for PM_2.5_ and PM_10_ with IHD hospitalizations at lag 0, prior to and after adjusting for the effect of OZONE across all seasons was shown in (Figure 3); and the corresponding results in the cold season was shown in (Figure 4).

The estimated effects of both PM_2.5_ and PM_10_ increased after factoring in the effect of OZONE in the models. The relationships between PM concentrations and IHD hospitalizations were almost linearly positive, and had no obvious threshold. The change of freedom per year for time trend (3 to 15) did not substantially affect the estimated effects (data not shown).

## 4. Discussion

This study demonstrated that the increase in IHD hospitalizations strongly coincided with the escalation of PM_2.5_ and PM_10_ concentrations in Shanghai, China during 2013–2014. In addition, the effect of PMs is strongest among males and patients in the 41 to 65 age group. The results also demonstrate the association between ambient PM exposure and increased rate of IHD hospitalizations.

Short-term exposure to PM has been linked to an increased occurrence of ischemic heart diseases. Exposure to air pollution may accelerate the progression of atherosclerosis, decrease plaque stability, reduce oxygen saturation and lead to hypoxemia, thereby increasing the cardiovascular burden and risk of IHD [21,22]. Studies have shown that the short-term effect of PM on IHD hospitalizations (including AMI episode, MI and angina) was predominantly prevalent during lag 0 to lag 7 [23,24,25,26]. Our results showed that the strongest effects of PM occurred on the current day of exposure (lag 0), which was consistent with previous studies performed in Shanghai, China [7,27]. This phenomenon could be attributed to the characteristics of local weather in Shanghai, while the presence of rain could reduce the concentrations of atmospheric pollutants and shorten the duration of PM effects on the risk of IHD.

Considering the fact that there may be multi-collinearity between PM and OZONE, we further assessed the role of OZONE in our model. After adjusting for the effects of OZONE, we found that there was a significant increase for both PM_2.5_ and PM_10_ on the rate of IHD hospitalization in both the cold seasons, as well as across all seasons. These results could provide important outlook for public health research and management. Notably, in order to prevent the increased risk of IHD due to PM pollution, increased public health awareness, for example, the wearing of masks and reduction in outdoor activities should be a priority. In addition, there should be increased government public health policies for decreasing air pollution, providing air purification in important areas such as schools, hospitals and factories, as well as advocating routine physical examinations for people at risk of high exposure to PMs. However, the association between PM and increased risk of cardiovascular events such as IHD remains controversial.

The relationship between PM exposure and myocardial infarctions were summarized in 2015 by Wang et al., who reviewed a total of 21 studies, and provided contrasting results [28]. Seven such studies did not find any association between PM and risk of MI, while the remaining 14 studies suggested the associations of MI with either PM_10_ or PM_2.5_, with varying lag days. This discrepancy might be due to the varying populations, climate differences and air pollution characteristics in each study. However, further study performed by Wang et al showed that there was no clear relationship between geographic situation, demographic characteristics and the effect of PM, indicating that differences in the effect of PM may be attributed to the individual’s exposure, susceptibility and response.

Studies have indicated that the effect of PMs on heart disease were greater in the cold season [18,29]. In Shanghai, the cold season has a relatively high average temperature (6.3 °C), and therefore people are more likely to open the windows and go outdoors [30]. In addition, the concentrations of PMs and gaseous pollutants were higher in the cold season than in the warm season (data not shown). Therefore, we analyzed the relationship between PMs and IHD hospitalizations in the cold season separately from the data across all seasons. The results depict that the effect of PM_2.5_ in cold season has been more robust than that in all seasons. A possible explanation is that low temperature can increase blood pressure and viscosity, which may lead to more frequent occurrences of cardiovascular attacks [31,32]. However, studies have shown contrasting results, for example, Zanobetti et al. found a stronger association between PM_2.5_ and MI hospitalizations during the warm season [26]. This phenomenon might be caused by different population exposure patterns, differences in population susceptibilities and lifestyles [30], for example, residents in Shanghai tend to use air conditioners more frequently in the warm season rather than the cold season, which may reduce the exposure to PMs.

In this study, we observed a greater effect of PMs on the risk of IHD in males compared to females, which may be partially explained by the fact that males are more likely to be exposed to air pollution due to the higher rate of outdoor work. We also observed a greater effect among people aged between 40 and 65, whereas no association was observed in people aged over 85. This result is inconsistent with previous studies, which showed that the influence of air pollution to the elderly was more detrimental [13,33,34]. It is presumed that elderly people, especially those aged over 85, are more sedentary and therefore more likely to stay at home or in hospital. On the other hand, the younger population is less likely to have a preexisting heart disease that confines them to a hospital, and therefore their chance of exposure to ambient air pollutants may be higher [35].

Our study has several strengths. First, this study is one of the few studies which reported the hazardous effects of air pollutants on IHD in a metropolitan area which is affected by slight to moderate levels of pollution. Second, our study consisted of a large sample size of 188,198 hospital admissions for IHD, and there were no missing data on air pollution and meteorological figures during the study period, thereby the PM data strongly supported the statistical findings. Finally, the air pollution and meteorological data acquired were from reliable sources; and the weather monitoring stations covered the entire area of Shanghai, which provided a great representation of the pollution situation within Shanghai.

Nevertheless, there are several limitations to our study. First, we could not measure the true PM exposure of individuals due to a lack of available data, such as the distance from the monitoring stations to the individual’s residence or workplace, as well as the unavailability of indoor exposure data. Therefore, each individual may have higher or lower exposure compared to our estimates. Second, due to the lack of the information on important risk factors that might affect the individual’s susceptibility to IHD, such as obesity, smoking habits, diet, exercise and other lifestyle factors, our study could not investigate the link between potentially sensitive subpopulations to particulate air pollution. Finally, although the classification of each patient was performed by the attending physician, there was potential for misclassification due to inaccuracies. Several studies have reported significant discrepancies between diagnosis and patient classifications; for example, Chiu et al. [36] found that 40% of patients identified by ICD codes as having a venous thromboembolism did not actually have venous thromboembolism. Further studies are required to provide more detailed assessment of clinical information, including imaging diagnosis and biochemical detection in order to minimize potential bias. Meanwhile, quality control studies should be performed to better assess the accuracy of the results.

## 5. Conclusions

Significant associations between PM_2.5_, PM_10_ and daily hospitalizations of IHD were identified in Shanghai, China during 2013–2014. Males and people aged between 40 and 65 are more susceptible to the effect of PMs. Our results provide new evidence of the effects of relatively low PM levels on the increased incidences of IHD and may suggest further investigation on specific cardiovascular diseases in Shanghai.

## Figures and Tables

**Table 1:** Summary of daily IHD hospitalizations in Shanghai in 2013–2014.

Group	All Seasons		Cold Season (November–April)	
	*n* (%)	Mean ± SD	*n* (%)	Mean ± SD
All	188,198	258.2 ± 103.6	97,041	268.8 ± 110.3
Gender				
Male	90,739 (48.2)	124.5 ± 50.4	46,821 (48.2)	129.7 ± 54.1
Female	97,459 (51.8)	133.7 ± 54.9	50,220 (51.8)	139.1 ± 57.9
Age (years)				
41–65	45,009 (23.9)	61.7 ± 27.7	22,769 (23.5)	63.1 ± 29.7
66–85	96,135 (51.1)	131.9 ± 52.5	49,719 (51.2)	137.7 ± 55.9
>85	47,054 (25.0)	64.6 ± 28.4	24,553 (25.3)	68.0 ± 30.2

IHD: ischemic heart disease.

**Table 2:** Summary of daily air pollutants and weather conditions in Shanghai in 2013–2014.

Variable	Frequency Distribution					Mean ± SD
	Minimum	25	50	75	Maximum	
All seasons						
Air pollutants (μg/m^3^)						
PM_10_	6.0	44.5	61.0	95.0	305.0	76.0 ± 47.5
PM_2.5_	8.0	30.0	46.0	70.0	255.0	56.3 ± 38.6
OZONE	13.0	72.0	96.0	121.0	302.0	101.5 ± 42.6
Weather conditions						
Temperature (°C)	−4.2	6.1	15.0	22.2	31.3	14.1 ± 9.2
Relative Humidity (%)	31.8	61.1	71.3	79.6	97.4	70.2 ± 12.6
Cold season						
Air pollutants (μg/m^3^)						
PM_10_	6.0	53.0	76.0	120.0	305.0	70 ± 44.6
PM_2.5_	8.0	39.0	58.0	87.0	255.0	91.9 ± 55.3
OZONE	13.0	60.0	78.0	100.5	206.0	81.4 ± 29.7
Weather conditions						
Temperature (°C)	−4.2	2.1	6.0	10.3	20.1	6.3 ± 5.3
Relative Humidity (%)	31.8	59.8	69.0	76.8	97.4	68.2 ± 13.2

**Table 3:** Percentage increase in IHD hospitalizations (95% CI) associated with a 10 μg/m^3^ increase in PM_2.5_ and PM_10_ concentrations at lag 0 by gender and age groups.

Group	All Seasons		Cold Season (November–April)	
	PM_2.5_	PM_10_	PM_2.5_	PM_10_
All	0.25 (0.10, 0.39) *	0.57 (0.46, 0.68) *	0.35 (0.19, 0.52) *	0.33 (0.19, 0.47) *
Gender				
Male	0.34 (0.14, 0.55) *	0.39 (0.21, 0.56) *	0.36 (0.12, 0.60) *	0.32 (0.12, 0.52) *
Female	0.29 (0.11, 0.48) *	0.28 (0.11, 0.45) *	0.35 (0.11, 0.58) *	0.35 (0.16, 0.55) *
Age (years)				
41–65	0.82 (0.52, 1.12) *	0.77 (0.52, 1.02) *	0.83 (0.49, 1.20) *	0.66 (0.37, 0.95) *
66–85	0.30 (0.11, 0.50) *	0.31 (0.14, 0.48) *	0.34 (0.10, 0.57) *	0.31 (0.12, 0.51) *
>85	−0.31 (−0.60, −0.02)	−0.03 (−0.27, 0.22)	−0.04 (−0.38, 0.29)	0.1 (−0.18, 0.38)

* *p* < 0.05.

## References

[1] Pun V.C., Yu I.T., Ho K., Qiu H., Sun Z., Tian L. (2014). Differential effects of source-specific particulate matter on emergency hospitalizations for ischemic heart disease in Hong Kong. Environ. Health Perspect..

[2] Liu J., Liu Y., Wang L., Yin P., Liu S., You J., Zeng X., Zhou M. (2015). The disease burden of cardiovascular and circulatory diseases in China, 1990 and 2010. Zhonghua Yu Fang Yi Xue Za Zhi.

[3] Wilson P.W.F., D’Agostino R.B., Sullivan L., Parise H., Kannel W.B. (2002). Overweight and obesity as determinants of cardiovascular risk—The Framingham experience. Arch. Internal Med..

[4] Dominici F., Peng R.D., Bell M.L., Pham L., McDermott A., Zeger S.L., Samet J.M. (2006). Fine particulate air pollution and hospital admission for cardiovascular and respiratory diseases. JAMA.

[5] Pope C.A., Muhlestein J.B., May H.T., Renlund D.G., Anderson J.L., Horne B.D. (2006). Ischemic heart disease events triggered by short-term exposure to fine particulate air pollution. Circulation.

[6] Xu M.M., Jia Y.P., Li G.X., Liu L.Q., Mo Y.Z., Jin X.B., Pan X.C. (2013). Relationship between ambient fine particles and ventricular repolarization changes and heart rate variability of elderly people with heart disease in Beijing, China. Biomed. Environ. Sci..

[7] Ye X.F., Peng L., Kan H.D., Wang W.B., Geng F.H., Mu Z., Zhou J., Yang D.D. (2016). Acute effects of particulate air pollution on the incidence of coronary heart disease in Shanghai, China. PLoS ONE.

[8] Nuvolone D., Balzi D., Chini M., Scala D., Giovannini F., Barchielli A. (2011). Short-term association between ambient air pollution and risk of hospitalization for acute myocardial infarction: Results of the cardiovascular risk and air pollution in Tuscany (RISCAT) study. Am. J. Epidemiol..

[9] Guo Y.M., Jia Y.P., Pan X.C., Liu L.Q., Wichmann H.E. (2009). The association between fine particulate air pollution and hospital emergency room visits for cardiovascular diseases in Beijing, China. Sci. Total Environ..

[10] Wolf K., Schneider A., Breitner S., Meisinger C., Heier M., Cyrys J., Kuch B., von Scheidt W., Peters A., Grp K.S. (2015). Associations between short-term exposure to particulate matter and ultrafine particles and myocardial infarction in Augsburg, Germany. Int. J. Hyg. Environ. Health.

[11] Evans J., van Donkelaar A., Martin R.V., Burnett R., Rainham D.G., Birkett N.J., Krewski D. (2013). Estimates of global mortality attributable to particulate air pollution using satellite imagery. Environ. Res..

[12] Guo Y.M., Barnett A.G., Zhang Y.S., Tong S.L., Yu W.W., Pan X.C. (2010). The short-term effect of air pollution on cardiovascular mortality in Tianjin, China: Comparison of time series and case-crossover analyses. Sci. Total Environ..

[13] Zhang P.F., Dong G.H., Sun B.J., Zhang L.W., Chen X., Ma N.N., Yu F., Guo H.M., Huang H., Lee Y.L. (2011). Long-term exposure to ambient air pollution and mortality due to cardiovascular disease and cerebrovascular disease in Shenyang, China. PLoS ONE.

[14] Xu M.M., Guo Y.M., Zhang Y.J., Westerdahl D., Mo Y.Z., Liang F.C., Pan X.C. (2014). Spatiotemporal analysis of particulate air pollution and ischemic heart disease mortality in Beijing, China. Environ. Health.

[15] Xie J., He M.Z., Zhu W.Y. (2014). Acute effects of outdoor air pollution on emergency department visits due to five clinical subtypes of coronary heart diseases in Shanghai, China. J. Epidemiol..

[16] Wang Z. (2012). Shanghai Statistical Yearbook 2012.

[17] Wang X., Chen R.J., Meng X., Geng F.H., Wang C.C., Kan H.D. (2013). Associations between fine particle, coarse particle, black carbon and hospital visits in a Chinese city. Sci. Total Environ..

[18] Kan H.D., London S.J., Chen G.H., Zhang Y.H., Song G.X., Zhao N.Q., Jiang L.L., Chen B.H. (2008). Season, sex, age, and education as modifiers of the effects of outdoor air pollution on daily mortality in Shanghai, China: The Public Health and Air Pollution in Asia (PAPA) study. Environ. Health Perspect..

[19] Zanobetti A., Schwartz J. (2005). The effect of particulate air pollution on emergency admissions for myocardial infarction: A multicity case-crossover analysis. Environ. Health Perspect..

[20] Peng R.D. (2008). Statistical Methods for Environmental Epidemiology with R.

[21] Routledge H.C., Manney S., Harrison R.M., Ayres J.G., Townend J.N. (2006). Effect of inhaled sulphur dioxide and carbon particles on heart rate variability and markers of inflammation and coagulation in human subjects. Heart.

[22] Bhaskaran K., Hajat S., Haines A., Herrett E., Wilkinson P., Smeeth L. (2009). Effects of air pollution on the incidence of myocardial infarction. Heart.

[23] Von Klot S., Peters A., Aalto P., Bellander T., Berglind N., D’Ippoliti D., Elosua R., Hormann A., Kulmala M., Lanki T. (2005). Ambient air pollution is associated with increased risk of hospital cardiac readmissions of myocardial infarction survivors in five European cities. Circulation.

[24] Lanki T., Pekkanen J., Aalto P., Elosua R., Berglind N., D’Ippoliti D., Kulmala M., Nyberg F., Peters A., Picciotto S. (2006). Associations of traffic related air pollutants with hospitalisation for first acute myocardial infarction: The HEAPSS study. Occup. Environ. Med..

[25] D’Ippoliti D., Forastiere F., Ancona C., Agabiti N., Fusco D., Michelozzi P., Perucci C.A. (2003). Air pollution and myocardial infarction in Rome—A case-crossover analysis. Epidemiology.

[26] Zanobetti A., Schwartz J. (2006). Air pollution and emergency admissions in Boston, MA. J. Epidemiol. Community Health.

[27] Dai J., Chen R., Meng X., Yang C., Zhao Z., Kan H. (2015). Ambient air pollution, temperature and out-of-hospital coronary deaths in Shanghai, China. Environ. Pollut..

[28] Wang X.M., Kindzierski W., Kaul P. (2015). Comparison of transient associations of air pollution and AMI hospitalisation in two cities of Alberta, Canada, using a case-crossover design. BMJ Open.

[29] Chen Y.C., Weng Y.H., Chiu Y.W., Yang C.Y. (2015). Short-term effects of coarse particulate matter on hospital admissions for cardiovascular diseases: A case-crossover study in a tropical city. J. Toxicol. Environ. Health Part A Curr. Issues.

[30] Qiu H., Yu I.T.S., Wang X.R., Tian L.W., Tse L.A., Wong T.W. (2013). Cool and dry weather enhances the effects of air pollution on emergency IHD hospital admissions. Int. J. Cardiol..

[31] Kyobutungi C., Grau A., Stieglbauer G., Becher H. (2005). Absolute temperature, temperature changes and stroke risk: A case-crossover study. Eur. J. Epidemiol..

[32] Hoffmann B., Luttmann-Gibson H., Cohen A., Zanobetti A., de Souza C., Foley C., Suh H.H., Coull B.A., Schwartz J., Mittleman M. (2012). Opposing effects of particle pollution, ozone, and ambient temperature on arterial blood pressure. Environ. Health Perspect..

[33] Zeka A., Zanobetti A., Schwartz J. (2006). Individual-level modifiers of the effects of particulate matter on daily mortality. Am. J. Epidemiol..

[34] Forastiere F., Stafoggia M., Berti G., Bisanti L., Cernigliaro A., Chiusolo M., Mallone S., Miglio R., Pandolfi P., Rognoni M. (2008). Particulate matter and daily mortality—A case-crossover analysis of individual effect modifiers. Epidemiology.

[35] Ueda K., Nitta H., Ono M. (2009). Effects of fine particulate matter on daily mortality for specific heart diseases in Japan. Circ. J..

[36] Chiu H.H., Whittaker P. (2013). Venous thromboembolism in an industrial north american city: Temporal distribution and association with particulate matter air pollution. PLoS ONE.

